# Shallow seamounts represent speciation islands for circumglobal yellowtail *Seriola lalandi*

**DOI:** 10.1038/s41598-021-82501-z

**Published:** 2021-02-11

**Authors:** Sven Kerwath, Rouvay Roodt-Wilding, Toufiek Samaai, Henning Winker, Wendy West, Sheroma Surajnarayan, Belinda Swart, Aletta Bester-van der Merwe, Albrecht Götz, Stephen Lamberth, Christopher Wilke

**Affiliations:** 1 Fisheries Management, Department of Environment, Forestry and Fisheries, Private Bag X2, Vlaeberg, 8018 South Africa; 2 Department of Biological Sciences, University of Cape Town, Private Bag X3, Rondebosch, 7701 South Africa; 3 Molecular Breeding and Biodiversity Group, Department of Genetics, Stellenbosch University, Private Bag X1, Stellenbosch, South Africa; 4 Oceans and Coasts, Department of Environment, Forestry and Fisheries, Private Bag X4390, Cape Town, Foreshore District, 8001 South Africa; 5 Department of Environmental Sciences, University of South Africa, PO Box 392, Unisa, 003 South Africa; 6 Elwandle Node, South African Environmental Observation Network (SAEON), PO Box 77000, Port Elizabeth, 6031 South Africa; 7 Zoology Department, Nelson Mandela Metropolitan University, Port Elizabeth, 6031 South Africa; 8 Department of Animal Sciences, Stellenbosch University, Private Bag X1, Stellenbosch, South Africa

**Keywords:** Ecology, Ecological genetics, Ocean sciences, Marine biology

## Abstract

Phenotypic plasticity in life-history traits in response to heterogeneous environments has been observed in a number of fishes. Conversely, genetic structure has recently been detected in even the most wide ranging pelagic teleost fish and shark species with massive dispersal potential, putting into question previous expectations of panmixia. Shallow oceanic seamounts are known aggregation sites for pelagic species, but their role in genetic structuring of widely distributed species remains poorly understood. The yellowtail kingfish (*Seriola lalandi*), a commercially valuable, circumglobal, epipelagic fish species occurs in two genetically distinct Southern Hemisphere populations (South Pacific and southern Africa) with low levels of gene-flow between the regions. Two shallow oceanic seamounts exist in the ocean basins around southern Africa; Vema and Walters Shoal in the Atlantic and Indian oceans, respectively. We analysed rare samples from these remote locations and from the South African continental shelf to assess genetic structure and population connectivity in *S. lalandi* and investigated life-history traits by comparing diet, age, growth and maturation among the three sites. The results suggest that yellowtail from South Africa and the two seamounts are genetically and phenotypically distinct. Rather than mere feeding oases, we postulate that these seamounts represent islands of breeding populations with site-specific adaptations.

## Introduction

Seamounts have long been known as aggregation sites for large pelagic fishes such as tuna, billfishes and sharks^1^. Phenotypic plasticity might explain observed differences in growth, maturation and reproduction related traits, depending on natural and fishing mortality, access to resources and environmental variation, as there has been little evidence of genetic isolation of seamount associated pelagic fish populations^2^, yet, recent literature has detected genetic structure in wide ranging large pelagic fishes^3^, questioning the current understanding of seamounts as mere feeding oases and navigation aids.

**Figure 1:**
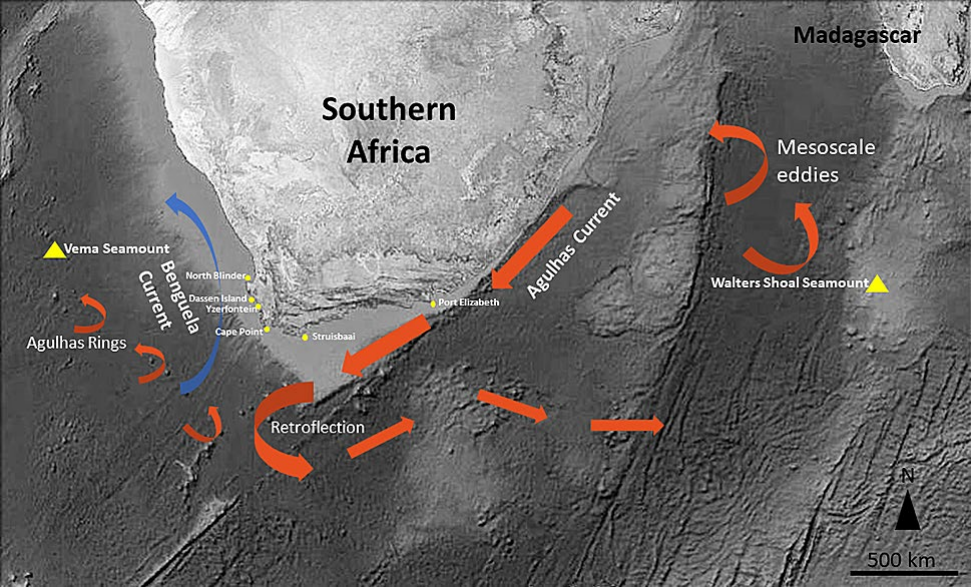
Map of southern Africa. The two shallow seamounts, Vema and Walters Shoal are indicated as yellow triangles, the South Africa sampling sites are indicated as yellow dots. Currents are schematised in form of arrows (red for warm, blue for cold currents). Background map created ex-novo by Dr Lauren Williams, Department of Environment, Forestry and Fisheries (DEFF), Oceans and Coasts Research, GIS Unit. Software ArcGIS version 10.3.1, licenced to DEFF.

Two oceanic seamounts that extend into the photic zone are known to exist around southern African waters, Vema (31° 41′ S, 8° 20′ E) in the southern Atlantic Ocean and Walters Shoal (33° 9′ S, 43° 49′ E) in the Southern Indian Ocean (Fig. 1). Both features harbour endemic species^4,5^ with a pelagic propagule phase, which suggests the existence of hydrographic mechanisms for larval retention. Yet, both are also home to common, widely distributed organisms such as the yellowtail kingfish, *Seriola lalandi* Valenciennes, 1833 (Carangidae). Yellowtail are circumglobal predators occurring in temperate and subtropical waters. Yellowtail occur in a variety of epipelagic habitats from the surf zone to the open ocean and aggregate around isolated features such as seamounts and reef pinnacles^6–8^. An important fisheries and aquaculture species throughout its distribution, aquaculture production of yellowtail in some countries generally outweighs that of wild commercial harvesting by up to 75%^9^, but wild stocks continue to support fisheries such as in South Africa^10,11^.

Yellowtail are pelagic nomads^10^ and their movement is strongly influenced by oceanographic conditions and prey availability^6,12,13^. Movements of up to 2960 km have been reported from tagging studies^14^ along with some site fidelity in larger specimens at certain sites, linked to availability of food and favourable environmental conditions, providing further support for a nomadic existence^6,15^.

In addition to the large range of individual adult yellowtail, this species has considerable dispersal potential for eggs, larvae and juveniles. Like many pelagic species, yellowtail are broadcast spawners that produce large numbers of pelagic eggs^16^ during multiple spawning events within a spawning season. Spawning in the Southern Hemisphere occurs in austral summer and has been reported from offshore reefs^6,12^, as well as within bays^17^. The larvae and juveniles are often associated with flotsam, which suggests potential for dispersal through ocean currents.

Despite the global distribution and long distance dispersal potential throughout all life history stages, phylogeographic analyses suggest significant spatial population differentiation on different geographic scales based on meristics and phylogenetics^18,19^.

In the current study we examined the genetic structure and life history characteristics, specifically diet and growth, of yellowtail associated with the two seamounts and from the South African continental shelf. Specifically we investigated the hypotheses that (1) Vema and Walters Shoal function as aggregation and feeding points of a panmictic yellowtail population distributed around Southern Africa (Oasis hypothesis) or (2) that Vema and Walters Shoal host populations of yellowtail genetically and phenotypically differentiated from each other and from coastal populations (Island hypothesis).

## Results

### Microsatellite data

Genetic diversity was determined in a total of 351 individuals from three main sampling regions using genotype data generated from six microsatellite markers. Population differentiation was also successfully assessed using F-statistics, multivariate and clustering analyses.

#### Genetic diversity

The software MICRO-CHECKER indicated that only two loci showed evidence of null alleles (Sdu29 and SduCA4j). These markers were included in subsequent analyses due to the limited number of microsatellite markers available in this study.

The average number of alleles per locus ranged from 2 (*Sdu10*) to 26 (*SduCA107*; Table S1 in Appendix S1). Allelic richness ranged from 2 (*Sdu10*) to 15.640 (*SduCA107*; Table S1 in Appendix S1), and average values were comparable between sampling population. Genetic diversity statistics as per locus was similar across sampling sites in terms of observed heterozygosity (Table S1 in Appendix S1). Among loci values of average observed heterozygosity ranged from Ho = 0.4167 at locus *Sdu46* to Ho = 0.9787 at locus *SduCA4j*.

Simulations of statistical power performed in POWSIM indicated that pairwise differentiation based on all 6 microsatellites at a level of F_ST_ > 0.010, was significant in 100% of the 1000 replicates for both chi-squared and Fisher’s exact test (Table S3 in Appendix S1). These results suggest that samples sizes and number of loci used here were large enough to detect genetic differentiation levels > 0.010.

#### Population differentiation

**Figure 2:**
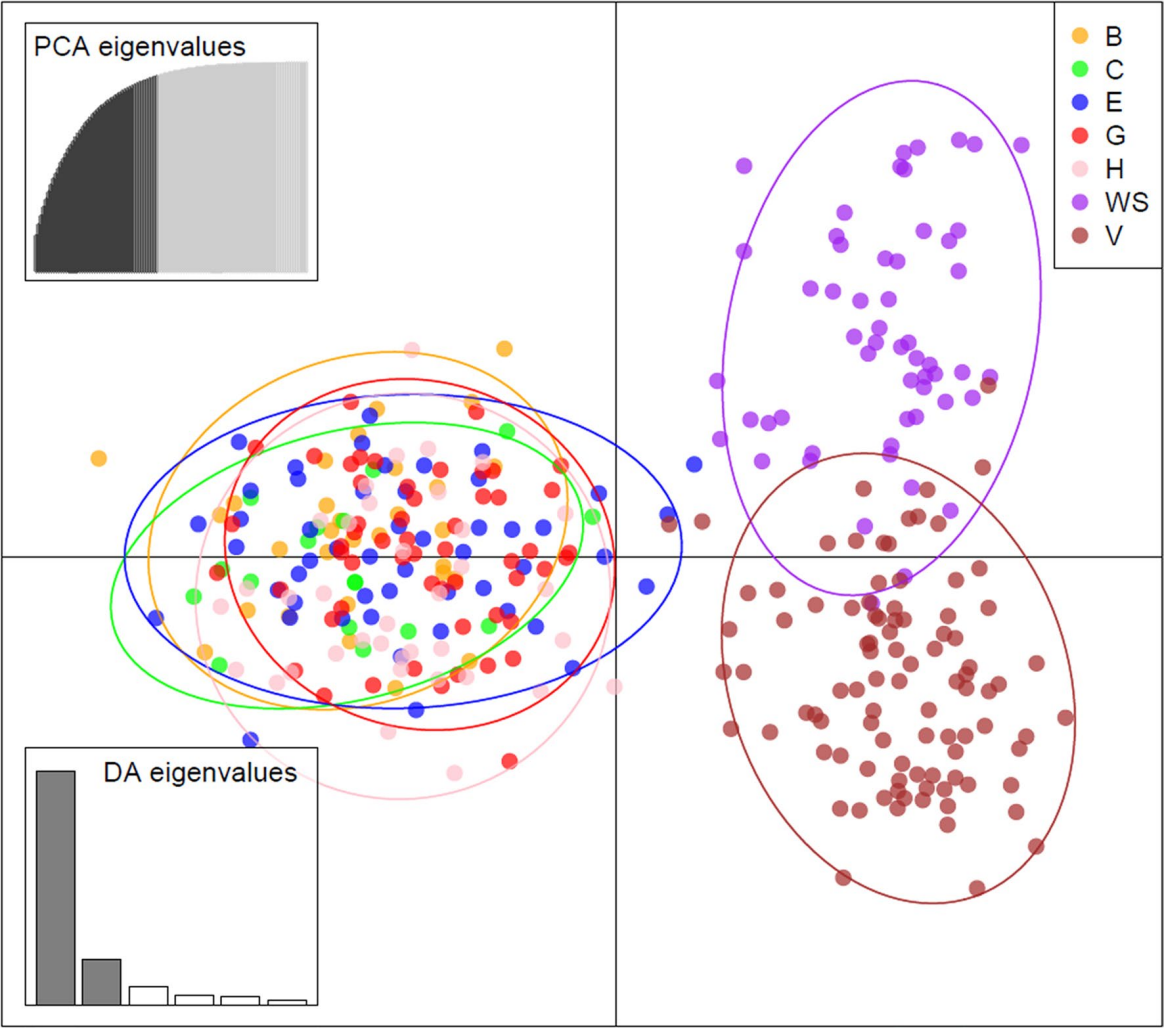
Discriminant analysis of principal components (DAPC) showing the principal components and 95% confidence ellipses of all *Seriola lalandi* samples: B—North Blinder, C—Yzerfontein, E—Cape Point, G—Struis Bay, H—Port Elizabeth. V—Vema, WS—Walters Shoal. PCA, principal component analysis; DA, discriminant analysis.

Overall, large genetic distances, and significant pairwise F_ST_ values were found between sampling sites from South Africa and the two seamounts; Vema and Walters Shoal (Table 1, Table S4 in Appendix S1). This strong genetic differentiation was supported by the large and significant G”_ST_ and Jost D_EST_ values found between the three main sampling regions (Table 2). Pairwise F_ST_ values between the five South African sampling sites were relatively low (Table 1), with the lowest between Cape Point, Struis Bay and Port Elizabeth. The F_ST_ values were only significant (p < 0.05) between the two South African sampling sites North Blinder and Yzerfontein. Pairwise F_ST_ values based on only the four loci devoid of null alleles showed a similar overall pattern with significant values only between sampling sites from South Africa and the two seamounts; Vema and Walters Shoal (Table S2 in Appendix S1). AMOVA revealed overall significant genetic structuring between South Africa and the two seamount populations (F_ST_ = 0.131, p = 0.000). Most of the total genetic variance was found between individuals within populations (86.88%) with very little variance distributed among populations within groups (1.47%). Results of the DAPC based on the 95% confidence ellipses showed a strong separation between individuals from South Africa and those from the two seamounts, with overlap mostly between individuals of the two seamounts (Fig. 2).

**Table 1:** Pairwise F_ST_ (1,000 permutations; above diagonal) values of seven *Seriola lalandi* populations.

	North Blinder	Yzerfontein	Cape Point	Struis Bay	Port Elizabeth	Walters Shoal	Vema
North Blinder	–	0.023*	0.015*	0.010*	0.018*	0.146*	0.163*
Yzerfontein		–	0.038*	0.029*	0.026*	0.164*	0.164*
Cape Point			–	0.007	0.009	0.143*	0.151*
Struis Bay				–	0.005	0.124*	0.138*
Port Elizabeth					–	0.138*	0.139*
Walters Shoal						–	0.043*
Vema							–

*Significant *F*_*ST*_* p-*values (*p* < 0.05) after Bonferroni correction.

**Table 2:** Pairwise G”_ST_ (999 permutations; below diagonal) and D_EST_ (999 permutations; above diagonal) values for the SA, Vema and Walters Shoal *Seriola lalandi* populations.

	South Africa	Walters Shoal	Vema
South Africa	–	0.543*	0.577*
Walters Shoal	0.612*	–	0.135*
Vema	0.644*	0.172*	–

*Significant *p-*values (*p* < 0.05) after Bonferroni correction.

Bayesian clustering analysis was used to infer the number of genetically distinct (ancestral) populations, assuming no prior information on the number of sampling locations. Based on ΔK, three clusters were detected and membership assignment plots (K = 3) indicated a clear separation between the South African, Vema and Walters Shoal seamount populations (Figure S1 in Appendix S1).

### Life history

#### Diet

All 54 (100%) stomach samples from Vema contained food. For the Walters Shoal specimens, only 28 (50%) of the 56 stomachs contained food whereas in the 62 South African samples, 47 (76%) contained food that could be partially identified.

The yellowtail diet at all three locations principally consisted of fish, crustaceans and cephalopods. In the stomachs of the Vema yellowtail, crustaceans had the highest frequency of occurrence (*FO)* with 81.5%, followed by fish with *FO* of 68.5% (Table S5 in Appendix S1). In the Walters Shoal yellowtail fish and crustaceans both had high *FO* of 67.9% and 64.3%, respectively.

Bony fish dominated the stomachs of the South African yellowtail with an *FO* of 74.19%. The yellowtail stomachs from Walters Shoal featured a larger variety of crustaceans compared to the Vema sample. The most frequent crustacean in the Vema sample was an euphausiid species which occurred in 81.5% of the stomachs. With 25%, *Lysiosquilla tredecimdentata* (mantis shrimp) had the highest *FO* in the Walters Shoal sample. Other crustacean species occurred in less than 15% of the stomachs at both seamount locations. The only crustacean group that made a significant contribution to the diet of yellowtail in South Africa were the crab megalopa larva (*FO* 19.35%).

Yellowtail stomachs from Walters Shoal contained a lower variety of fish species with only three families identified, as compared to the Vema samples, which contained six different families and South Africa with four different families. No common fish species were found in yellowtail stomachs at the three locations. Cephalopods were found in 28.6% of the Walters Shoal stomach samples compared to 18.5% and 9.68% for the Vema and South African stomach samples, respectively. The cephalopods were difficult to identify even to family level, however, two families (Histioteuthidae, Ommastrephidae) from one order (Teuthida) were identified in the Vema diet while one family (Octopoteuthidae) and two orders (Octopoda, Teuthida) were found in the Walters Shoal sample. Molluscs did not feature in the Walters Shoal diet and were of negligible importance in the diet of the Vema and South African yellowtail with a *FO* of 7.4% and > 2%, respectively.

#### Age and growth

**Figure 3:**
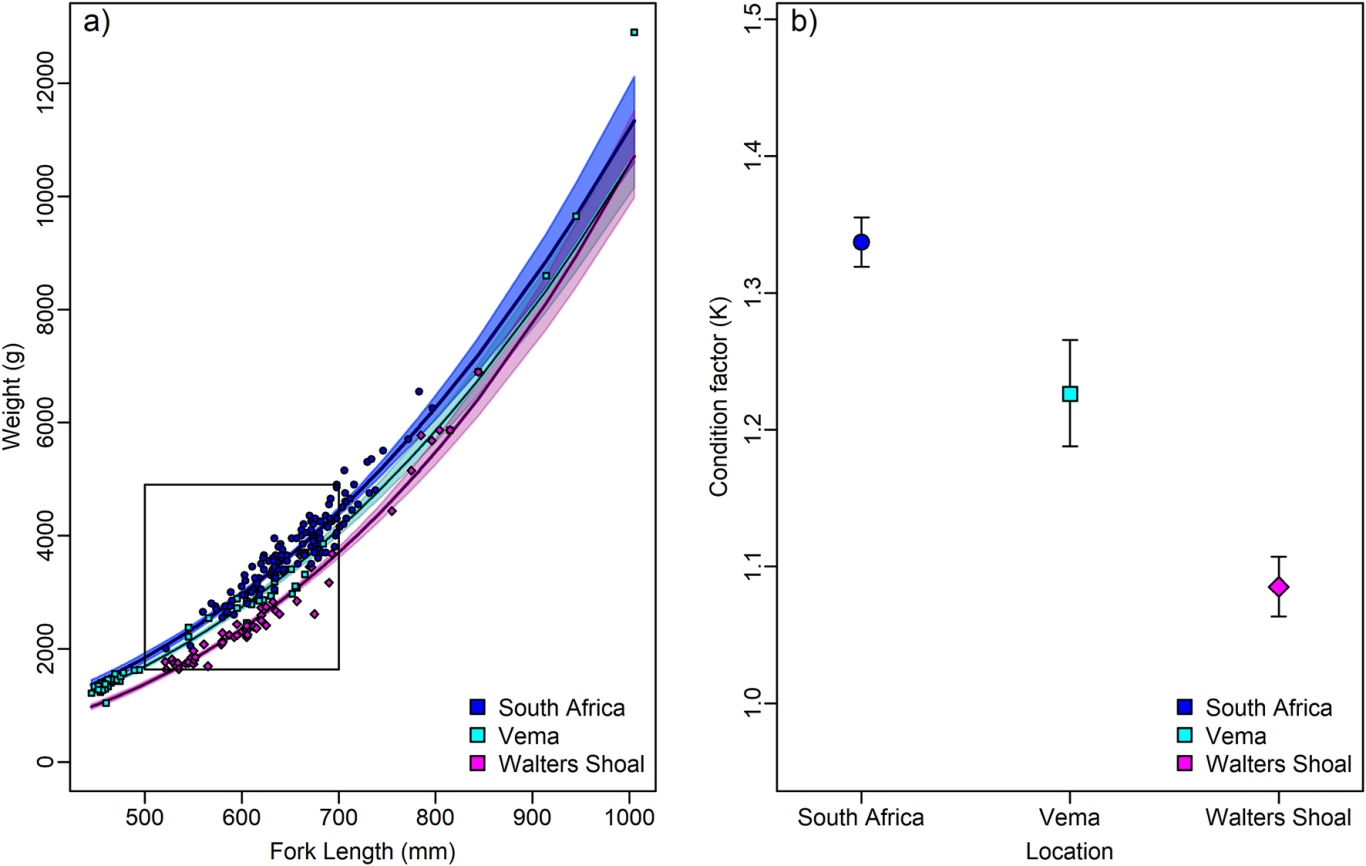
(**a**) Observed (symbols) and predicted (solid lines) length–weight relationships and (**b**) expected mean values of the condition factor *K* for yellowtail from sampling localities from South Africa, Vema and Walters Shoal sampling localities. The box highlights the size classes of 500 to 700 mm FL fish that were well represented for each sampling localities and used calculate the condition factor. Colour coded translucent areas (**a**) and error bars (**b**) denote the 95% confidence intervals the respective sampling locality. Differences in the colour gradients of shaded areas (**a**) result from overlaps in 95% confidence areas.

Yellowtail sampled at Vema displayed a broad size range from 445 mm FL and a weight of 1.220 g to 1.005 mm FL and a weight of 12.900 g. The South African yellowtail ages ranged from 490 mm FL and a weight of 2.000 g to 916 mm FL and a weight 6.550 g. The fish sampled at Walters Shoal ranged from 521 mm FL and a weight of 1.772 g to 844 mm FL and a weight of 6.894 g. Length–weight relationships differed among the locations (Fig. 3a), resulting in highly significantly differences in the respective condition factors *K* (Fig. 3b), as judged by an one-way ANOVA applied specimens of 500–700 mm *FL* (*F* = 142.6*, df* = 178, p < 0*.*05). The condition factor was highest for Vema followed by South Africa and Walters Shoal.

**Figure 4:**
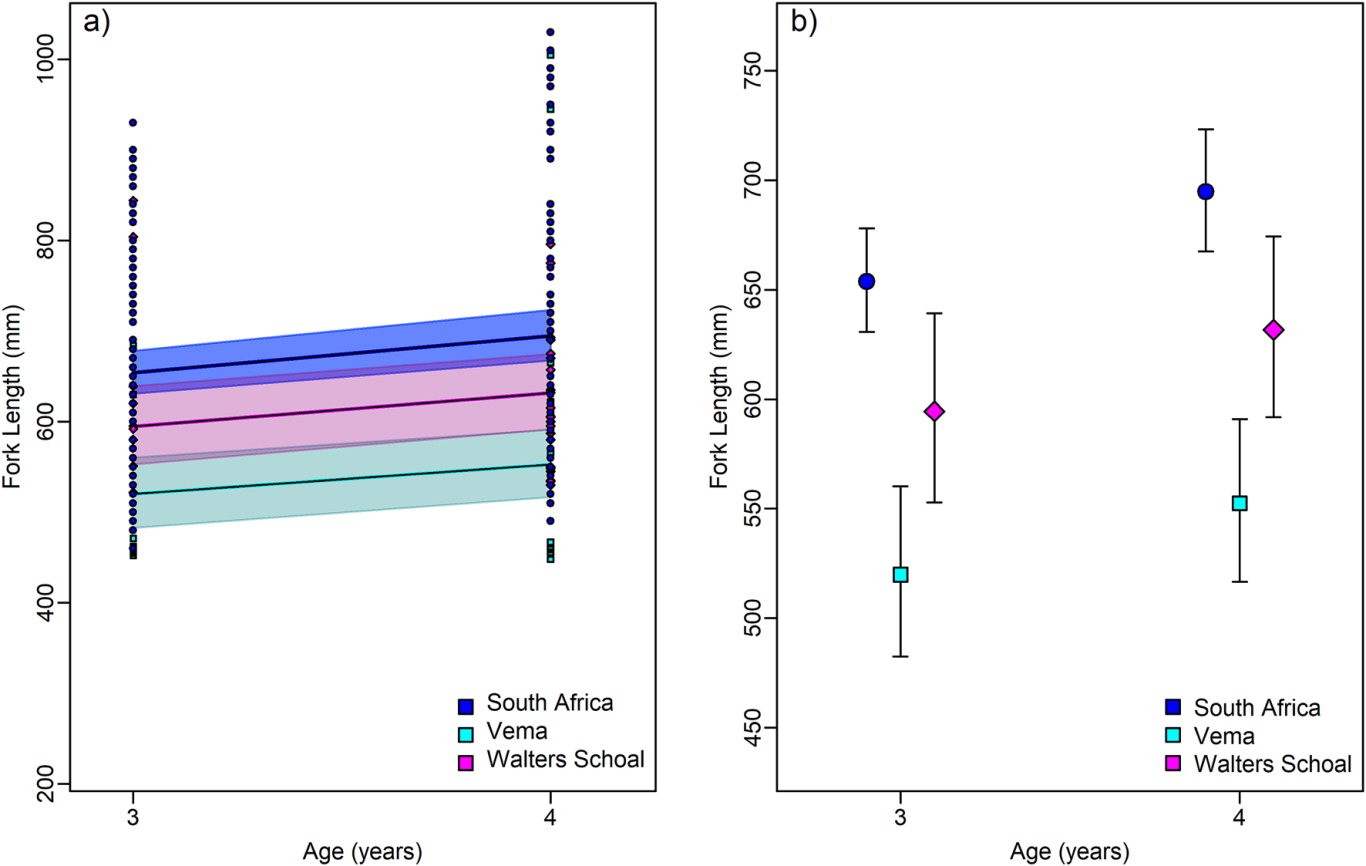
(**a**) Observed (symbols) and predicted (solid lines) lengths-at-age of Age-3 and Age-4 yellowtail from South Africa, Vema and Walters Shoal sampling localities. Coloured coded translucent areas (**a**) and error bars (**b**) denote the 95% confidence intervals the respective sampling locality. Difference in the gradients shades (**a**) result from overlaps in 95% confidence areas. Differences in the colour gradients of shaded areas (**a**) result from overlaps in 95% confidence areas.

Age readings were accepted for 83.3% of the individuals from the Vema sample, 73% of the South African specimens and 87.5% of the Walters Shoal sample, with APE and CV values of 15.5% and 14.8% (Vema), 16.2% and 21.7% (South Africa), and 10.5% and 11.5% (Walters Shoal), respectively. Age readings ranged from 2 to 7 years for all subsamples combined, but only the year classes 3 and 4 had large enough sample sizes (Vema = 31, SA = 162, WS = 33) for a meaningful comparison. The two-way ANOVA showed that there was a highly significant difference in length-at-age among the three locations (*F* = 20.08, *df* = 222, *p* < 0.001) (Fig. 4a). Three and four year old fishes were largest in South Africa, followed by Walters Shoal and Vema (Fig. 4b).

#### Sex ratio and gonad development

The Vema sample consisted of 54 individuals, 19 of which were males ranging from 445 to 914 mm fork length (FL), 25 females ranging from 452 to 1005 mm FL and the remainder were unidentifiable. The sex ratio of males to females was found to be 1:1.32. The number of mature females in the sample was 20 (80%), the smallest mature females were 452 mm FL in both stage 2 and 3 classes while the largest mature female was recorded at 1,005 mm FL (stage 3). The number of mature males in the sample was 18 (95%), the smallest mature male was recorded at 445 mm FL (stage 2) while the largest mature male was found to be 914 mm FL stage 3.

In the 51 South African samples, the smallest mature male was 520 mm FL. All males above 820 mm FL were mature. The smallest mature female observed in this study was 520 mm FL. All females above 780 mm FL were mature. The sex ratio of males to females was found to be 1:1.15.

**Figure 5:**
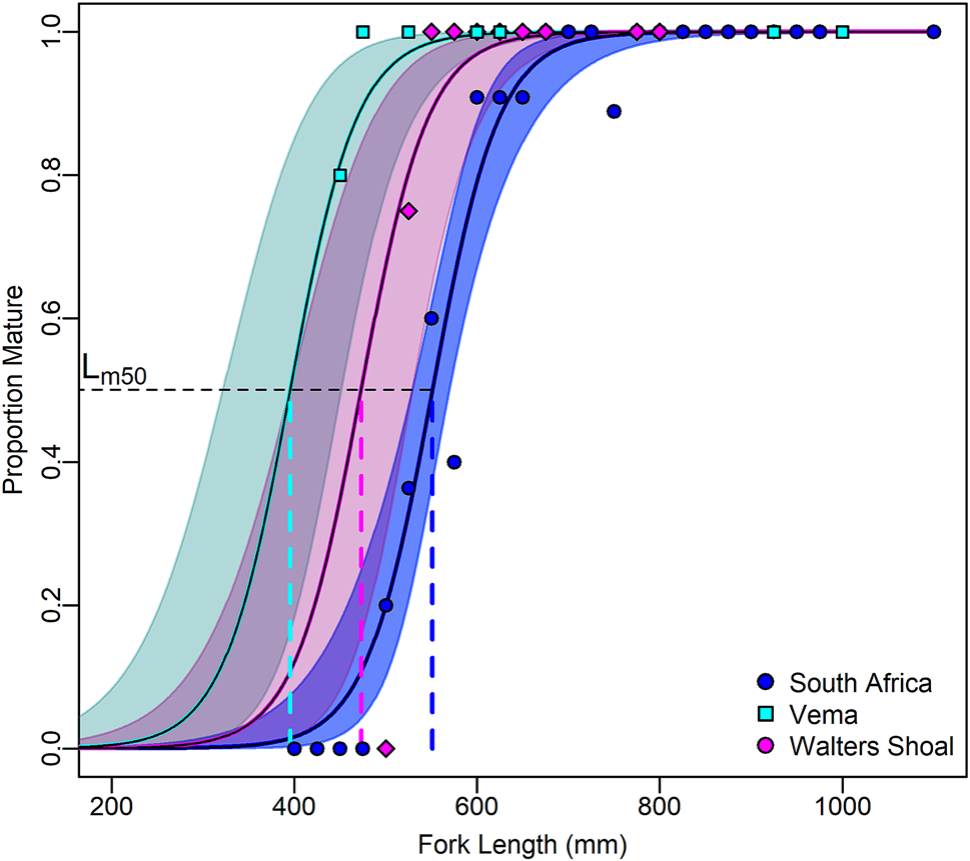
Logistic maturity ogives showing observed (symbols) and predicted (solid lines) -lengths-at-maturity for female yellowtail from South Africa, Vema and Walters Shoal sampling localities. Colour-coded translucent areas denote the 95% confidence intervals the respective sampling locality. Colour-code dashed lines depict the estimated lengtht-at-50%-maturity (*L*_*m50*_) for each locality. Differences in the colour gradients of shaded areas result from overlaps in 95% confidence areas.

Of the 56 yellowtail from Walters Shoal, 22 were males ranging from 530 to 844 mm FL and 34 were females ranging from 521 to 815 mm FL. The smallest mature female was 521 mm FL (stage 3) and the largest mature female was 815 mm FL, also stage 3. The number of mature males in the sample was 10 (45%). The smallest male was 530 mm FL (stage 2) and the largest male was 844 mm FL (stage 2). Analysis of deviance found that locality explained significant variation in female length-at-maturity (*p* < 0.001), with length-at-50%-maturity (*L*_*m50*_) estimated to occur significantly smaller at Vema with 395.1 mm FL (*p* < 0.001) and Walter Shoal with 473.2 mm FL (*p* < 0.01) than in South Africa with 551.2 mm FL (Fig. 5).

## Discussion

Despite the species’ movement behaviour and dispersal potential, our results revealed the following: (1) the existence of genetically and phenotypically distinct populations of yellowtail associated with the South African continental shelf and the two seamounts in the Atlantic and Indian oceans; and (2) supports the ‘Island’ hypothesis (seamounts are regarded as solitary islands, where speciation occurs^20^) as opposed to the ‘Oasis’ hypothesis^21^.

The microsatellite data support the existence of three genetically distinct groups of yellowtail across the sampling regions with little connectivity between South Africa and the two seamounts. This implies the three main geographic sampling regions either serve as reservoirs for larval or egg retention and/or adult individuals return to the regions where they were spawned.

Overall estimates of genetic variability were significantly different between the three main sampling regions and significant pairwise F_ST,_ G”_ST_ and D_EST_ comparisons were observed between all South African sampling sites and the two seamounts. Both multivariate (DAPC) and Bayesian clustering analysis supported the existence of three distinct genetic clusters corresponding to South Africa, Walters Shoal and Vema seamount.

Marine animal populations are linked to each other via dispersal of individuals as eggs, larvae, juveniles or adults, a process known as connectivity. The extent of dispersal and connectivity is determined by several factors, including oceanographic and hydrological features as well as the reproductive strategy of the species^22^. Retention and dispersal of sedentary species is largely a function of passive drift of eggs and larvae, limiting connectivity between seamounts^23,24^. On the other hand, mobile species such as yellowtail are theoretically able to move between seamounts as juveniles and during their reproductive phase^25^.

A higher level of connectivity and thus gene flow was evident between the two seamounts. The passive dispersal of eggs and larvae (17 days of preflexion) and the movement of juveniles via flotsam^26^ could result in dispersal over hundreds of kilometers along major ocean currents, resulting in high gene flow over a large distance. Walters Shoal is occasionally swept by mesoscale eddies originating from the East Madagascar Current, one of the Agulhas Current’s tributaries. The Agulhas Current flows in south-westerly direction along the edge of the continental shelf of the African continent. Near 36°S at the Agulhas Bank, it separates from the continental shelf and turns eastwards in an anticyclonic loop, named the Agulhas retroflection by Bang^27^. It then flows back into the South Indian Ocean as the Agulhas Return Current. Periodically the loop of the retroflection forms an eddy, which is ejected into the South Atlantic Ocean^28^. The Vema seamount is in the path of these Agulhas rings^29,30^. These oceanographic features provide a mechanism for passive transport of propagules and juveniles from Walters Shoal to Vema, bypassing the South African coastline. This would also explain some of the genetic admixture observed around Cape Point which is the closest 'exit point' for passively drifting objects before the uptake into the Benguela jet.

Connectivity between seamounts is a key element affecting the degree of isolation or similarity of seamount populations. Studies on the genetic population connectivity among commercially important seamount and slope fish populations have however provided varied results. Most studies report genetic homogeneity between seamount and continental and oceanic island slopes including species such as the northern Pacific pelagic armourhead (*Pseudopentaceros wheeleri*), the alfonsino (*Beryx splendens*), black oreo (*Allocyttus niger*), smooth oreo (*Pseudocyttus maculates*), orange roughy (*Hoplostethus atlanticus*) and offshore rockfish (*Pontinus kuhlii*)^31–36^. On the contrary, genetic heterogeneity or differentiation between seamount and continental populations has been identified in only a few species of fish, (e.g., between the continental margin of Europe, and the Azores Islands on the Mid-Atlantic Ridge in blue-mouth red fish *Helicolenus dactylopterus*)^37^. Mitochondrial DNA and microsatellite analyses of black sea bream (*Pagellus bogaraveo*) populations between slopes of the Azores Islands in the North Atlantic revealed low to moderate (but significant) genetic differentiation with evidence for a recent bottleneck, perhaps due to the impacts of fishing^38^. In the patagonian toothfish (*Dissostichus eleginoides*), although the mitochondrial DNA marker 12S rDNA failed to reveal genetic structure^39^, differences in microsatellite frequencies were evident between seamount and non-seamount populations.

The observed genetic differences among yellowtail are concomitant with differences in life history and diet. Albeit based on small, single sampling events, due to the difficulty of obtaining samples from such remote locations, our results suggest that the three samples differentiate by condition factor, diet, length at age and size at maturity. Walters Shoal fish have the lowest weight-at-length, resulting in a significantly lower condition factor, followed by Vema and South Africa. This might be a result of a combination of several factors: The higher temperature in the subtropical environment, which increases metabolic rates and the lower food availability around the seamounts when compared to the South African coast.

The Benguela current system is one of the largest upwelling zones in the world with highly productive waters^40^. The waters around Walters Shoal are oligotrophic and relatively unproductive when compared to the Vema and the South African populations. The observed difference in diet, with piscivorous feeding dominant in the South African fish and crustacean dominated diet around the seamounts supports the explanation for the difference in condition factor. Yellowtail are known to be opportunistic generalist feeders preying on small pelagic fish, crustaceans and squid^6,41^. Small pelagic shoaling species such as sardine (*Sardinops sargax*) have been reported to be the preferred food source around coastal South Africa in previous studies^41^ and studies suggest that small pelagic fish are of higher nutritional value than demersal fish and crustaceans^42^, the latter two being more prevalent in the diet of the seamount associated samples.

We found significant differences in growth rates, represented as length-at-age, as well as in the length-at-maturation among the three sampling localities. Life history theory assumes adaptive plasticity in age and size at maturity^43^. A trade-off between early maturation, with fewer numbers of offspring with shorter generation length, and later maturation, with increased viability and number of offspring and longer generation length, depending on mortality and access to resources can explain inter- and intra-species variation in these traits^44,45^.

Life history theory predicts trade-offs among growth, maturation and survival^45,46^. Delayed maturity is expected to reduce sub-adult mortality and promotes sustained higher growth performances, as no energy needs to be invested into gonad development. As female body mass and fecundity increase exponentially with size, the advantage of this strategy has been suggested to increase the total egg production capacity (fitness) per spawner^47^. The largest size at maturity found in Walters Shoal yellowtail is in line with the assumptions that maturation can be delayed when offspring viability needs to be enhanced. Walters Shoal, due to the high temperature and oligotrophy of the surrounding waters possibly represents an environment that requires more larval resilience. Vema yellowtail on the other hand matured early and were of the smallest size at age. As food restrictions are unlikely an issue at Vema, the observed pattern might be best explained by increased fishing mortality. Selection for early maturation as well as smaller size at age is thought to be common as fisheries often remove larger fish^48^. Vema has been under heavy fishing pressure since its discovery in 1957 and a population of the spiny lobster *Jasus tristani* had been fished to local extinction within less than ten years of its discovery^49,50^. Reported catch of yellowtail at Vema by tuna directed commercial fishing vessels is substantial (DEFF, unpublished data) and IUU fishing is thought to be considerable. The fish sampled off South Africa have the largest length at age and intermediate size at 50% maturity, representing an intermediary position in the trade-off envelope with highest growth due to the most consistent food availability, combined with moderate fishing mortality.

## Conclusion

This study represents one of only a few genetic studies to include samples from these remote seamounts, and is the first for an actively dispersing species. The findings suggest that seamounts can harbour unique populations of commercially fished species distinct from populations associated with continental shelf areas. Differences in life history patterns can largely be explained by phenotypic plasticity related to different environmental conditions and mortality rates, but in combination with the observed genetic differences our findings are in agreement with the ‘Island’ Hypothesis that yellowtail exist in genetically and phenotypically distinguishable populations. Our findings imply that these populations might need separate assessment, management and conservation.

## Methods

### Localities and sampling

Vema seamount (31° 41′ S, 8° 20′ E) is situated in the South Atlantic Ocean approximately 950 km west of South Africa (Fig. 1). Rising approximately 5000 m from the surrounding plane, it summits at 22 m below the surface^50^. Walters Shoal (33° 9′ S, 43° 49′ E) seamount is an isolated seamount found in the Indian Ocean on the Madagascar Ridge. Its location is approximately 1700 km east of South Africa and 850 km south of Madagascar (Fig. 1). It peaks at 18 m below the surface of the water with an area of approximately three nautical miles across the shallowest plateau-like part of the seamount^5^. Biogeographically, the area can be described as warm-temperate to subtropical.

The South African temperate coast is influenced by two major current systems, the Benguela, a broad, northward flow along the West Coast that forms the part of the South Atlantic Subtropical Gyre, and the Agulhas, a narrow, warm, swift south-westerly flow along the South African East and South Coast. The two systems are the principal drivers of productivity in the coastal and shelf waters due to upwelling and subsequent plankton blooms and they facilitate the transport of propagules of marine organisms in South African waters^51^.

Samples from Vema were obtained in May 2015 from a pelagic fishery operating occasionally around the seamount. South African yellowtail samples were collected by hook and line fishing during dedicated research outings during austral summer 2011 and 2012 between Dassen Island and Struis Bay. Further genetic samples were obtained from across the South African distributional range of *S. lalandi* by the South African Department of Environment, Forestry and Fisheries (DEFF) land based observer programme (see fig. 1 in Swart^18^). Walters Shoal was sampled during a dedicated, month-long scientific expedition on the *RV Algoa* in May 2014 when fish were caught by hook and line. Only material from dead animals was used for further analyses. All collections were made under a “permit for the purposes of a scientific investigation or practical experiment in terms of section 79 of the Marine Living Resources Act 1998” issued by the then Department of Agriculture, Forestry and Fisheries, South Africa. All methods were carried out in accordance with relevant guidelines and regulations.

The samples were frozen until processing in the laboratory. Upon defrosting all specimens were measured to the nearest mm fork length (FL) and total length (TL) and weighed to the nearest gram. Gonads were weighed to the nearest gram and sex and stage of maturation was determined. Stomachs were staged in terms of fullness and weighed to the nearest gram after which the stomach contents were removed and frozen. Both otoliths were removed from the fish, cleaned and kept dry for further processing. In addition, fin clips were taken and stored in 90% ethanol for subsequent genetic analyses. Information on sample sizes for individual analyses are included in the methodology sections.

### DNA extraction

In this study, a total of 201 individuals from South Africa (SA) as well as samples from Vema Seamount (V = 95) and Walters Shoal (WS = 55) were included for microsatellite genotyping. The South African samples comprised Port Elizabeth (H) = 37, Struis Bay (G) = 55, Cape Point (E) = 50, Yzerfontein (C) = 29 and North Blinder (B) = 30) (Fig. 1, also see fig. 1 in Swart^19^). The South African sample sites were included in a previous study investigating the global and regional population structure of the species^19^.

All samples were morphologically identified by the sample collectors and preserved in 95% ethanol. Genomic DNA extraction was carried out according to the cetyl trimethyl ammonium bromide (CTAB) protocol^56,57^ with minor modification.

### PCR amplification and microsatellite genotyping

Six microsatellite markers previously published for *S. dumerili*^52^ were successfully amplified by PCR^53^ and genotyped in the seven sampling populations (Vema, 5 sampling populations in SA, Walters Shoal). Fragment analysis was performed by capillary electrophoresis on the ABI 3100 automated sequencer. Alleles were scored using MICROSATELIGHT v1^54^.

### Microsatellite genotype analyses

#### Population diversity

MICRO-CHECKER v2.2.3 was used to test for the presence of null alleles, genotyping errors or other reasons for deviations from HWE, such as heterozygote deficiency^55^.

The population genetic variation for *S. lalandi* samples was characterised for the two seamounts and the five locations along the South African coastline^19^. Genetic diversity estimates of allele frequencies, observed- (Ho) and unbiased expected heterozygosity (He) were calculated with GENETIX v4.03^56^. Fstat v2.9.3^57^ was used to determine the number of alleles per locus and allelic richness based on a minimum sample size of 29. The inbreeding coefficient, F_IS_ was calculated by Fstat and deviations from Hardy–Weinberg equilibrium (HWE) were tested with ARLEQUIN.

The statistical power of the microsatellites to differentiate populations were evaluated and conducted in POWSIM v4.1^19,58^. This analysis simulates multiple populations that have diverged to predefined true levels of divergence (F_ST_), and determines the power of a dataset with sample sizes, number of loci, and allele frequencies equal to the present study to differentiate populations. Simulations included true levels of divergence (F_ST_) that ranged from 0.002 to 0.049, and significance for both Fisher’s exact test and χ^2^ tests from 1000 replicates. Three simulations were conducted with varying effective population size (Ne = 100, 200, 500 and 2000).

#### Population structure

Pairwise F_ST_^59,60^ (with Bonferroni correction at the 5% nominal level) and Nei’s (1978) genetic distances^61^ were calculated with GENEPOP v4.0^62^ using 1000 permutations. In addition, pairwise G”_ST_^63^ and Jost D_EST_^64^ were calculated with GenAlEx 6.5^65,66^ (999 permutations) between the three main sampling regions. To visualise genotypic partitioning, discriminant analysis of principal components (DAPC)^67^ was carried out using ADEGENET^68^ in R 3.0.2^69^. To test for population differentiation, a hierarchical AMOVA was carried out in ARLEQUIN^19^.

Bayesian clustering analysis of populations was performed with STRUCTURE v2.1^70^. For the analysis on the entire dataset, number of clusters (K) was tested between 1 and 6. For each K, 10 runs were performed consisting of 10 million Markov chain Monte Carlo (MCMC) iterations with a burn-in of 100,000. K was calculated assuming admixture and correlated allele frequencies^18,71^. The statistic ΔK calculated by STRUCTURE HARVESTER v0.6^72^ was used to estimate the true number of clusters^73^. For the selected K value, the individual membership coefficient was assessed to infer the probability of a certain individual belonging to a specific cluster. CLUMPP v1.1 was used to average the runs and produce the admixture (Q) matrix^74^. DISTRUCT v1.1 was implemented to visualise the membership coefficients of the individuals within each population^75^.

### Life history

#### Diet

Stomachs (Sample sizes: V = 54; SA = 62; WS = 56) were defrosted and the contents extracted by opening the stomach and squeezing out the contents. The numbers of stomachs with food present or absent were recorded to determine the coefficient of repletion (percentage of stomachs containing food). Upon visual inspection, the contents were separated into individual components where possible. For the identification of fish, cephalopods and mollusc prey items, the otoliths and beaks and shells were collected, respectively, after initial visual examination of partially digested specimens was deemed inconclusive. Only, crustaceans could be identified from largely complete exoskeletons. Where necessary, specimens were examined under a dissecting microscope and identified to the lowest possible taxon using identification guidebooks and experts. Due to the variation in stomach fullness and regurgitation rates among sites, comparisons involving prey weight such as the index of relative importance for specific prey species were not feasible. The frequency of occurrence (*FO*) for each prey item was calculated as the number of stomachs that particular prey item was found in divided by the total number of stomachs examined.

#### Age and growth

All fish sampled (345) were measured to the nearest mm fork length (*FL*) and weighted to nearest gram. Morphometric relationships were determined by plotting the *FL* of the fish against their corresponding weights. The values for the growth parameters *a* and *b* were determined by log transformation and linear regression of the form:

1 $$log\left(W\right)=log\left(a\right)+FL*b$$

The condition factor (*K*) was determined as follows^76^:

2 $$K=100\times W/{\left(FL\right)}^{3}$$

The majority of specimens (74%) fell within 500 and 700 mm size classes, which were representatively sampled for all three locations, whereas smaller fish and very large fish (> 800 mm) were underrepresented in samples from the two seamounts. For appropriate statistical comparison of differences in *K*, a one-way ANOVA (Sample sizes: V = 57; SA = 132; WS = 56) was therefore only applied to specimens from the representatively sampled 500–700 mm size classes across the three locations.

The right otolith of a pair was used for age determination. Whole otoliths set in black plastic trays were viewed and imaged under a Nikon dissecting microscope at 8–16 × magnification with reflected light. Images were taken and three readers examined the samples independently after which the data was collated and compared. The accepted age was the age for which two or three readers were in agreement. As the small sample sizes and the incomplete size spectrum of the samples precluded fitting a meaningful growth model, the sizes of yellowtail with 3 and 4 growth rings (i.e. 3 and 4 years old, V = 31, SA = 162, WS = 33) were compared among locations by fitting a two-way ANOVA with the covariates *Age* and *Location*.

#### Sex ratio and gonad development

Visual inspection of the gonads provided the sex and the stage of sexual maturity (Sample sizes: V = 54; SA = 51; WS = 56). The proportion of sexually mature males and females was determined by considering any individual at stage 2 or higher as mature. The statistical analysis) only included female data due to their higher relevance to spawning output of the populations. The maturity status of each female was transformed into a binary response variable by assigning 0 for immature and 1 for mature or maturing females. Due to the expected binominal distribution of the binary response, a GLM (Generalized Linear Model) with binomial distribution and a logit link function was fitted to test for statistical differences in length-at-maturity among the three locations, where location was treated as factor. Analogous to an ANOVA, analysis of deviance based on was conducted to test if the factor location explained significant variation in the data as judged by χ^2^-tests of changes in total deviation explained.

## Supplementary Information

Supplementary Information
