# Supplementary material for: Shallow seamounts represent speciation islands for circumglobal yellowtail Seriola lalandi
Source: Sci Rep. 2021 Feb 11;11:3559. doi: 10.1038/s41598-021-82501-z (PMC7878507; doi:10.1038/s41598-021-82501-z)
Supplement: Supplementary file 1 — Supplementary Information [file 41598_2021_82501_MOESM1_ESM.pdf]

Supplementary Information

Shallow seamounts represent speciation islands for circumglobal yellowtail *Seriola lalandi*

Sven Kerwath[1,2,8,*], Rouvay Roodt-Wilding[3], Toufiek Samaai[2,4], Henning Winker[1], Wendy West[1], Sheroma Surajnarayan[5], Belinda Swart[3], Aletta Bester-van der Merwe[3], Albrecht Götz[6], Stephen Lamberth[1,7], Christopher Wilke[1]

[1]*Fisheries Management, Department of Environment, Forestry and Fisheries, Private Bag X2, Vlaeberg 8018, South Africa.*

[2]*Department of Biological Sciences, University of Cape Town, Private Bag X3, Rondebosch 7701, South Africa.*

[3]*Molecular Breeding and Biodiversity Group, Department of Genetics, Stellenbosch University, Private Bag X1, Stellenbosch, South Africa.*

[4]*Oceans and Coasts, Department of Environment, Forestry and Fisheries, Privat Bag X4390, Capetown, Foreshore District, 8001, South Africa.*

[5]*Department of Environmental Sciences, University of South Africa, PO Box 392.*

[6]*Elwandle Node, South African Environmental Observation Network (SAEON), PO Box 77000, Port Elizabeth, 6031, South Africa.*

[7]*Zoology Department, Nelson Mandela Metropolitan University, Port Elizabeth, 6031, South Africa.*

[8]*Department of Animal Sciences, Stellenbosch University, Private Bag X1, Stellenbosch, South Africa.*

***Corresponding author.** e-mail: SvenK@daff.gov.za

a)

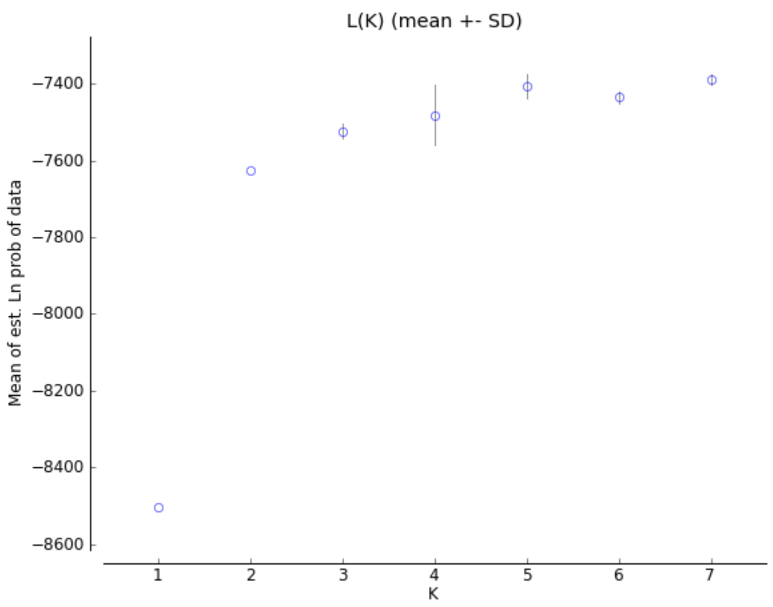

b)

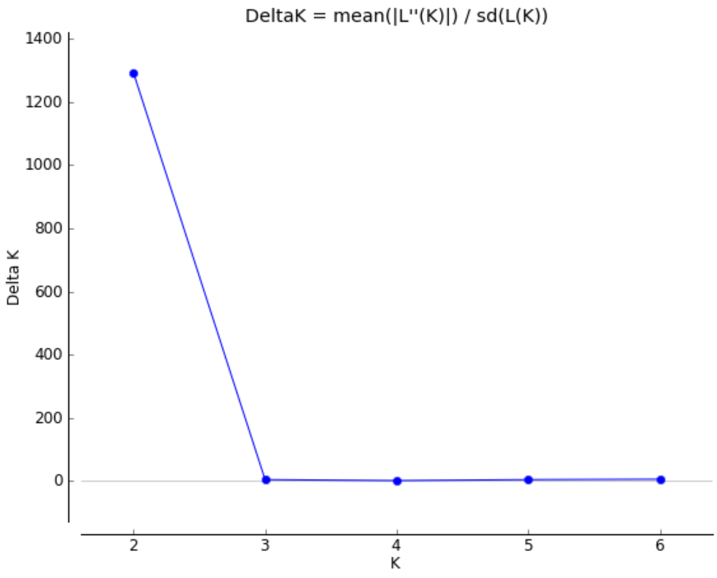

Figure S1

(a) The posterior probability of the data, L(*K*) for each *K* and (b) Δ*K* as a function of *K* following Evanno *et al.* (2005) for the sampling localities.

Table S1

Summary of genetic variation for six analysed microsatellite loci at seven *Seriola lalandi* sampling sites.

		**Loci**						
**Samples**		*Sdu10*	*SduCA107*	*Sdu29*	*Sdu32*	*Sdu46*	*SduCA4j*	Mean
North	*He*	0.4753	0.8978	0.8377	0.8491	0.4561	0.8068	0.7205
Blinder	*Ho*	0.5556	0.8148	0.6552	0.8462	0.4444	0.6071	0.6539
(B)	*Na*	2	17	10	14	5	7	
N = 30	*Ar*	2.000	15.398	9.035	12.550	4.918	6.921	
	F_{IS}	-0.150	0.111	0.235	0.023	0.044	0.264	0.111
Yzerfontein	*He*	0.4898	0.8937	0.7997	0.8025	0.4297	0.8469	0.7104
(C)	*Ho*	0.4286	0.7778	0.5357	0.6500	0.4167	0.8571	0.6110
N = 29	*Na*	2	15	8	10	4	12	
	Ar	2.000	13.727	7.553	10.000	3.950	11.153	
	F_{IS}	0.143	0.148	0.346	0.215	0.052	0.006	0.160
Cape Point	*He*	0.4231	0.8869	0.8546	0.8629	0.5826	0.7689	0.7298
(E)	*Ho*	0.4615	0.8367	0.6800	0.8182	0.5278	0.9787	0.7172
N = 50	*Na*	3	20	15	17	6	6	
	Ar	2.513	13.718	11.162	13.433	5.437	5.655	
	F_{IS}	-0.078	0.067	0.214	0.063	0.108	-0.263	0.029
Struis Bay	*He*	0.4783	0.8946	0.8566	0.8350	0.6234	0.8178	0.7510
(G)	*Ho*	0.6667	0.7551	0.8400	0.8200	0.6000	0.9600	0.7736
N = 55	*Na*	2	18	16	16	6	9	
	Ar	2.000	13.169	11.472	11.649	5.479	7.842	
	F_{IS}	-0.385	0.166	0.029	0.028	0.050	-0.164	-0.020
Port Elizabeth	*He*	0.5019	0.8885	0.8355	0.8484	0.5696	0.8333	0.7462
(H)	*Ho*	0.4167	0.8056	0.9714	0.7778	0.6400	0.8485	0.7433
N = 37	*Na*	3	20	13	16	5	9	
	Ar	2.806	15.267	11.134	12.932	4.800	8.848	

	F_{IS}	0.184	0.107	-0.149	0.097	-0.103	-0.003	0.019
Walters Shoal	*He*	0.487	0.917	0.868	0.802	0.691	0.731	0.750
(WS)	*Ho*	0.764	0.891	0.778	0.982	0.891	0.963	0.878
N = 55	*Na*	2	18	14	11	5	11	
	Ar	2.00	14.640	10.430	8.730	4.973	8.484	
	F_{IS}	-0.563	0.039	0.114	-0.216	-0.281	-0.309	-0.162
Vema	*He*	0.488	0.930	0.846	0.805	0.613	0.865	0.753
(V)	*Ho*	0.590	0.798	0.785	0.947	0.695	0.947	0.794
N = 95	*Na*	4	26	16	16	7	15	
	Ar	2.588	15.970	10.627	10.842	5.612	9.769	
	F_{IS}	-0.204	0.147	0.077	-0.172	-0.128	-0.130	-0.049
Overall	*He*	0.626	0.934	0.894	0.858	0.764	0.873	
	Ho	0.579	0.811	0.764	0.871	0.649	0.911	
	Na	7	36	22	25	10	17	
	Ar	3.358	16.831	12.477	12.241	6.859	10.540	
	F	0.080	0.134	0.147	-0.014	0.152	-0.042	
	PHWE	0.000	0.000	0.000	0.000	0.000	0.000	

Ho = observed heterozygosity; *He* = expected heterozygosity; *Na* = number of alleles; *Ar* = allelic richness, F_{IS} = inbreeding coefficient. PHWE = probability of Hardy-Weinberg equilibrium

Table S2

Pairwise F_{ST} (1,000 permutations) for 4 loci.

	North Blinder	Yzerfontein	Cape Point	Struis Bay	Port Elizabeth	Walters Shoal	Vema
North Blinder	-						
Yzerfontein	0.004	-					
Cape Point	0.011	0.023	-				
Struis Bay	0.010	0.018	0.001	-			
Port Elizabeth	0.015	0.021	0.006	0.001	-		
Walters Shoal	0.191*	0.199*	0.184*	0.161*	0.174*	-	
Vema	0.195*	0.203*	0.186*	0.166*	0.176*	0.008*	-

*Significant F_{ST} *p*-values (p <0.05) after Bonferroni correction

Table S3

Statistical power for detecting various true levels of population differentiation (F_{ST}) by means of χ^2 and Fisher's exact test when using the present loci, allele frequencies, and sample sizes. The power is expressed as the proportion of simulations that provide statistical significance at the 0.05 level.

F_{ST}	χ^2	Fisher
F_{ST} = 0.049	1.000	1.000
F_{ST} = 0.025	1.000	1.000
F_{ST} = 0.010	1.000	1.000
F_{ST} = 0.002	0.989	0.989

Table S4

Nei's genetic distances between the seven *S. lalandi* sampling localities for all loci combined.

	North Blinder	Yzerfontein	Cape Point	Struis Bay	Port Elizabeth	Walters Shoal
North Blinder	-					
Yzerfontein	0.069	-				
Cape Point	0.026	0.105	-			
Struis Bay	0.016	0.077	0.018	-		
Port Elizabeth	0.027	0.046	0.013	0.016	-	
Walters Shoal	0.816	0.938	0.832	0.694	0.779	-
Vema	0.985	0.921	0.939	0.802	0.782	0.143

Table S5

Frequency of occurrence (FO) of prey items from *S. lalandi* stomachs sampled at Vema, South Africa and Walters Shoal.

Group	Class	Order	Family	Species	Vema	South Africa	Walters Shoal
Fish					**68.5**	**74.2**	**67.9**
	**Actinopterygii**				24.1		14.3
		**Beloniformes**					
			**Hemiramphidae**			3.23	
			**Scomberesocidae**				
				**Scomberesox saurus**		4.84	
		**Clupeiformes**					
			**Clupeidae**				
				**Sardinops sagax**		16.13	
		**Myctophiformes**					
			**Myctophidae**		16.7		7.1
				**Diaphus lucidus**	7.4		
				**Diaphus problematicus**	1.9		
				**Gymnoscopelus piabilis**	1.9		
				**Hygophum proximum**	1.9		
				**Myctophum nitidulum**	1.9		
				**Myctophum phengodes**	3.7		
				**Notoscopelus spp**			3.6
				**Symbolophorus barnadi**	1.9		
				**Symbolophorus boops**	1.9		
		**Perciformes**			11.1		7.1
			**Acanthuridae**		1.9		
			**Carangidae**		3.7		3.6

	Decapterus macrosoma		1.61	
	Trachurus delagoa	3.7		
	Trachurus trachurus		11.3	3.6
	Gempylidae	1.9		3.6
	Labridae	3.7		
	Wrasse spp	1.9		
	Nomeidae	5.6		
	Cubiceps capensis	1.9		
	Cubiceps careulus	3.7		
	Sparidae		1.6	
	Boopsoidea inornata		1.61	
	Scorpaeniformes		6.45	
	Unknown species	29.6	50.0	28.6
Molluscs		**7.4**	**1.6**	**0**
	Bivalvia	1.9		
	Ostreoida			
	Ostreoidae	1.9		
	Mytilida			
	Mytilidae			
	Perna perna		1.61	
	Gastropoda	5.6		
	Littorinimorpha			
	Atlantidae	5.6		
Cephalo-pods		**18.5**	**9.7**	**28.6**
	Cephalopoda	7.4		14.3
	Myopsida			
	Loliginidae			

				**Loligo vulgaris**		4.84	
		**Octopoda**					3.6
			**Argonautidae**				
				**Argonauta argo**		1.61	
		**Teuthida**			7.4		3.6
			**Histioteuthidae**		5.6		
			**Octopoteuthidae**				
				**Octopoteuthis rugosa**			3.6
			**Ommastrephidae**		1.9		
	**Unknown species**				13.0	5.4	14.3
Crusta-ceans					**81.5**	**21.0**	**64.3**
	**Malacostraca**				**81.5**		**46.4**
		**Amphipoda**			11.1	3.2	7.1
			**Hyperiidae**				3.6
				**Themisto gaudichaudi**		3.23	
			**Platyscelidae**				
				**Platyscelus ovoides**	7.4		
		**Decapoda**			16.7	19.4	17.9
			**Paguroidea**				
				**Hermit crab larvae**	3.7		
			**Portunidae**				7.1
				**Portunid crab**			3.6
				**Portunus pelagicus**			7.1
		**Euphausiacea**					
			**Euphausiidae**		81.5	3.2	
				**Euphausia lucens**		1.61	
		**Isopoda**					14.3

	**Mysida**			
	**Mysidae**			
	**Gastrosaccus psammodytes**		1.61	
	**Stomatopoda**			
	**Lysiosquillidae**			
	**Lysiosquilla tredecimdentata**			25
	**Odontodactylidae**			
	**Odontodactylus scyllarus**			10.7
	**Unknown species**		1.6	10.7
Other		**7.4**	**1.6**	**39.3**
	**Polychaeta**		**1.61**	
	**Unknown species**	**3.7**		**17.9**

Table S6

Macroscopic staging of female and male gonads according to Dunn (2014). For histological descriptions of female and male gonads see Dunn (2014).

Maturity Stage	Macroscopic Condition
FEMALES	FEMALES
(1) Immature	Ovary lobes are oval in shape and thread-like at less than 60 mm in length.
(2) Active	Ovary lobes rounded in shape and much larger in size than in immature fish.
(3) Developing	Ovary lobes rounded and greater than 60 mm in length.
(4) Late Developing	Ovary large and yellow in colour with oocytes becoming visible.
(5) Ripe	Ovary firm and large, mature and hydrated oocytes are visible.
(6) Spent	Ovary flaccid, decreased in size and bloody, no oocytes are visible.
MALES	MALES
(1) Immature	Thread-like lobes.
(2) Active	Elongated, oval to triangular in cross-section.
(3) Mature	Elongated, larger in size and triangular in cross-section, pink tinge.
(4) Ripe	Large and soft, creamy white in colour, milt present in main duct.
